# NLRP1 inflammasome contributes to chronic stress-induced depressive-like behaviors in mice

**DOI:** 10.1186/s12974-020-01848-8

**Published:** 2020-06-08

**Authors:** Ao-Qi Song, Bo Gao, Jun-Juan Fan, Ya-Jing Zhu, Jun Zhou, Yu-Ling Wang, Li-Zhong Xu, Wen-Ning Wu

**Affiliations:** 1 Department of Pharmacology, School of Basic Medical Sciences, Anhui Medical University, Hefei, 230032 People’s Republic of China; 2 Department of Pharmacy, Xi’an Chest Hospital, Xi’an Jiaotong University, Xi’an, 710100 People’s Republic of China; 3 Key Laboratory of Anti-inflammatory and Immunopharmacology, Anhui Medical University, Hefei, 230032 People’s Republic of China

**Keywords:** NLRP1 inflammasome, MDD, Chronic stress, BDNF, CXCL1/CXCR2

## Abstract

**Background:**

Major depressive disorder (MDD) is a highly prevalent psychiatric disorder, and inflammation has been considered crucial components of the pathogenesis of depression. NLRP1 inflammasome-driven inflammatory response is believed to participate in many neurological disorders. However, it is unclear whether NLRP1 inflammasome is implicated in the development of depression.

**Methods:**

Animal models of depression were established by four different chronic stress stimuli including chronic unpredictable mild stress (CUMS), chronic restrain stress (CRS), chronic social defeat stress (CSDS), and repeat social defeat stress (RSDS). Depressive-like behaviors were determined by sucrose preference test (SPT), forced swim test (FST), tail-suspension test (TST), open-field test (OFT), social interaction test (SIT), and light-dark test (LDT). The expression of NLRP1 inflammasome complexes, BDNF, and CXCL1/CXCR2 were tested by western blot and quantitative real-time PCR. The levels of inflammatory cytokines were tested by enzyme-linked immunosorbent assay (ELISA) kits. Nlrp1a knockdown was performed by an adeno-associated virus (AAV) vector containing Nlrp1a-shRNA-eGFP infusion.

**Results:**

Chronic stress stimuli activated hippocampal NLRP1 inflammasome and promoted the release of pro-inflammatory cytokines IL-1β, IL-18, IL-6, and TNF-α in mice. Hippocampal Nlrp1a knockdown prevented NLRP1 inflammasome-driven inflammatory response and ameliorated stress-induced depressive-like behaviors. Also, chronic stress stimuli caused the increase in hippocampal CXCL1/CXCR2 expression and low BDNF levels in mice. Interestingly, Nlrp1a knockdown inhibited the up-regulation of CXCL1/CXCR2 expression and restored BDNF levels in the hippocampus.

**Conclusions:**

NLRP1 inflammasome-driven inflammatory response contributes to chronic stress induced depressive-like behaviors and the mechanism may be related to CXCL1/CXCR2/BDNF signaling pathway. Thus, NLRP1 inflammasome could become a potential antidepressant target.

## Background

Major depressive disorder (MDD) is a highly prevalent psychiatric disorder affecting more than 300 million people worldwide that is characterized by depressed mood, ruminative thoughts, anhedonia, cognitive dysfunction, vegetative symptoms, and even a high suicidal tendency [1–4]. This disorder impairs the quality of life and causes a burden to patients, their families, and society [5, 6]. MDD has been predicted to be the leading cause of global burden of disease by 2030 [7]. Despite there being a variety of available antidepressant, more than one-third of patients are refractory to these drugs [8, 9]. Thus, a deeper understanding of pathogenic events and molecular changes that occur during the course of depression is needed to develop more efficacious treatments for these patients.

Accumulating eviden,ce from clinical and experimental studies has shown that inflammation plays a critical role in depression [10, 11]. Patients with depression have increased pro-inflammatory cytokines in blood, such as IL-1β, IL-6, TNF-α, and other acute phase proteins, C-reactive protein (CRP) [12–14]. The levels of the pro-inflammatory cytokines are higher in patients with treatment-resistant depression (TRD) [15]. Also, increased pro-inflammatory cytokines genes were found in the frontal cortex of subjects with depressio n[16]. Anti-inflammatory treatments can produce anti-depressive effects, and antidepressant can attenuate the expression of pro-inflammatory cytokines in depression [17–19]. Similarly, experimental studies have also shown that depressive-like behaviors are associated with increased inflammatory mediators in the brain [20]. Administration of pro-inflammatory cytokines produces depressive-like behaviors whereas anti-inflammatory approaches can ameliorate depressive-like behaviors [21–23]. Inflammation has been considered important components of the pathogenesis of depressive disorders.

Inflammasomes are muti-protein complexes that consist of a cytosolic pattern-recognition receptor (a member of nucleotide oligomerization domain (NOD)-like receptor (NLR) family or HIN domain-containing (PYHIN) family), an adaptor known as apoptosis-associated speck-like protein containing a caspase-activating recruitment domain (ASC) and pro-caspase-1 [24]. Activation of inflammasomes can promote the production and release of pro-inflammatory cytokines such as IL-1β and IL-18 [25, 26]. As a critical platform regulating immune inflammatory reactions, inflammasomes-driven inflammatory responses have been reported to be involved in many neurological diseases, including CNS infections, brain injury and neurodegenerative diseases such as Alzheimer’s diseases (AD), Parkinson’s diseases (PD), and multiple sclerosis [27]. NLRP1 and NLRP3 belong to NLR family and are both expressed in CNS [28]. NLRP3, mostly expressed in microglia, has been reported to be engaged in animal models of depression and patients with MDD [28–31]. Unlike NLRP3, NLRP1 is mainly presented in neurons and dominantly implicated in neuronal injury pathologies and cognitive impairment [28, 32, 33], which is a core feature of subjects with depression. Also, recent studies showed that chronic unpredictable mild stress (CUMS) increase the expression of NLRP1 inflammasome complexes in animal models [34, 35]. However, the definitive role of NLRP1 inflammasome in depression remains unclear.

In this study, we established depression models of mice via four different chronic stress stimuli, which are major risk factors of MDD [36], and investigated the role of NLRP1 inflammasome in depression. Our results showed that stress stimuli activated NLRP1 inflammasome-driven inflammatory signaling pathway, while blockage of NLRP1 inflammasome activation ameliorated stress-induced depressive-like behaviors.

## Methods

### Animals

Male C57BL/6 mice (7–8 weeks old, 20–23 g) and male CD-1 mice (13–15 weeks) were obtained from the Experimental Animal Center of Anhui Medical University. Animals were kept under standard specific pathogen free conditions at an appropriate temperature (22 ± 2 °C) and humidity of 60% under a 12 h light/dark cycle. Food and water were available ad libitum. All animal procedures were approved by the Committee for Experimental Animal Use and Care of Anhui Medical University.

### Chemicals

Primary antibodies of NLRP1, caspase-1, and BDNF were purchased from Abcam (San Francisco, CA, USA), while antibody of ASC was purchased from Santa Cruz Biotechnology (Santa Cruz, CA, USA). Horseradish peroxidase-conjugated secondary antibodies were purchased from Santa Cruz Biotechnology (Santa Cruz, CA, USA). Other general agents were commercially available.

### Chronic stress procedures

Chronic unpredictable mild stress (CUMS) was performed as previously reported with minor modifications [37]. The mice were isolated in the individual cages and exposed to the following stressors: (1) warm swimming at 45 °C for 10 min; (2) cold swim at 4 °C for 10 min; (3) water deprivation for 24 h; (4) food deprivation for 24 h; (5) the cage tilting for 24 h (45 °C); (6) tail pinching for 5 min; (7) reversal of the light/dark cycle for 24 h; (8) wet pad for 24 h; (9) restraint stress for 2 h; (10) shaky cage 1 time/s for 10 min; (11) no bedding for 24 h; (12) tail hanging for 10 min. These stressors were randomly executed once daily for 6 weeks. Chronic restrain stress (CRS) was performed as previously reported with minor modifications [37]. The mice were placed in a 50 ml conical tube (with 0.5 cm air holes for breathing) for 2 h every day for a total of 10 days. Chronic social defeat stress (CSDS) as performed as previously reported with minor modifications [37]. The C57BL/6J was exposed to a different CD1 aggressor mouse for 5–10 min each day. Following contact, Non-touchable isolation was given within 24 h. This protocol was processed for 10 days and each mouse was subjected to expose to the different CD1 mice within 10 days. On day 11, defeated mice were subjected to the SI test. Repeat social defeat stress (RSDS) was performed as previously described with minor modifications [38]. The cages of mice (three per cage) were exposed to an aggressive intruder male CD1 mouse for 6 consecutive nights between 5:00 and 7:00 P.M. During each cycle, we observed submissive behavior including upright posture, fleeing and crouching to ensure that the mice showed subordinate behavior. If the invader did not attack them within 5–10 min, then a new invader was introduced. At the end of the 2 h period, the invader was removed and the cages of mice (three per cage) were left undisturbed until the following day when the modeling procedure was repeated. Different invaders were used on consecutive nights. During the social defeat stress, if the mice are injured, the wound was treated with antiseptic spray at the end of each stress session. The mice will be removed from the experiment when the wound becomes too severe (open wounds larger than 1 cm). All mice in the control group were raised normally.

### Sucrose preference test (SPT)

One day after last stress session, the sucrose preference test was used to observe anhedonic-like behavior. All mice were deprived of food and water for 12 h before the SPT began. Then, the mice were given two standard drinking bottles, one containing 1% sucrose and the other with tap water for a period of 24 h. The positions of these two bottles were switched every 6 h to avoid side preferences. The sucrose and water intakes were measured when the SPT was over, and the sucrose preference rate was presented as percentage of sucrose intake/sucrose intake plus water intake.

### Open-field test (OFT)

One day after last stress session, the open-field test was performed under the bright interior lighting to evaluate the locomotor and exploratory behavior of the mice. The open-field apparatus (96 × 96 × 50 cm) was divided into 9 equal squares. Firstly, the mice were placed in the center area, allowing them adapting to the environment for 2 min. Then the total movement distance, the time spent in center, and the number of crossing the center were recorded over a 3 min period. At each stress session, the floor surfaces and walls of the OFT apparatus were thoroughly cleaned with a 75% ethanol to abolish any sign of olfactory cues.

### Tail-suspension test (TST)

Two days after last stress session, the mouse’s tail was suspended from a plastic rod with tape (5 cm wide) and put the mouse in a head-down position. We controlled the distance from the ground to the head of the mouse to about 50 cm. The experiment lasted 6 min. During the 6 min period of the test, each mouse was allowed to adapt for 2 min after being suspended, the amount of time the animals still stayed was recorded during the remaining 4 min.

### Forced swim test (FST)

Three days after last stress session, forced swim test was carried out as a highly reliable test for evaluating depressive-like behavioral state. The mice were placed individually into a clear glass cylinder (25 cm in diameter) filled with 30 cm depth of water (maintained at 24 ± 1 °C). The time of immobility was defined as immobile time spent by the mice floating in the water with no active movements but movements that were necessary to keep their heads above the water. The formal test was monitored by a vidicon. Each mouse was allowed to adapt for 3 min to the conditions, then the total immobility time was recorded over the following 4 min.

### Social interaction test (SIT)

One day after last stress session, social interaction test was conducted in a white plastic open box (area size 45 × 45 × 45 cm). The test consists of two parts. The activities of animals are monitored by video surveillance software. First, an area of 24 × 14 cm is defined as a social contact area on one side of the open box. A small compartment (area size 8 × 6 cm) was separated from the social contact area by a transparent plastic spacer for preventing CD1 mice. The plastic separator has a number of small holes that allow the C57BL/6J mice to smell the strange CD1 mice in the compartment. Next, in the absence of CD1 mice in the compartment (target absent phase), the C57BL/6J mice were placed in the central area of the open box and allowed to move freely for 2.5 min. At the same time, statistics were performed on the times the animals entered the social contact area during the 2.5 min test period (part 1 test). The open box was cleaned after the end of the first part of the test. In the second part of the test (target present stage), an unfamiliar CD1 mouse that has never contacted with the C57BL/6J mice is placed in the compartment. The C57BL/6J mice were again placed in an open box and recorded the times they entered the social contact area during the 2.5 min. The social contact rate of each animal C57BL/6J was calculated as follows: SI ratio = target time (in interaction zone)/no target time (in interaction zone). According to the residence time of the C57BL/6J mice in the contact area and the social contact ratio, the experimental mice subjected to the CSDS stress treatment can be preliminarily grouped. The mice, whose social interaction ratio is less than 1, were classified as stress-sensitive mice, and the mice with social interaction ratio more than 1 were considered as resistant mice.

### Light-dark test (LDT)

The LDB is a wooden rectangular box that is divided into two regions. The smaller area is a black box (area size 18 × 27 × 27 cm), the inner wall is black, and the top cover is unlit; the larger area is the bright box (area size 45 × 27 × 27 cm), the inner wall is white, and the top cover is illuminated by LED lights. One day after last stress session, the mice were placed in the experimental field before the test to adapt to the environment about 3 min. At the beginning of the test, the mice were placed in the middle of the dark box and then allowed to move freely for 5 min each time. The free activity of the mice at the experimental site was observed and recorded by the monitoring system automatically. After each experiment, wiping the perimeter wall and floor with 75% alcohol to prevent animal residues from affecting the next test results. During the process of the mice in the bright and dark box, the system can automatically judge and record these animal behaviors. Data includes: light compartment duration (uTIME), the total time that the mice were in the bright box with activity or staying; dark compartment duration (pTIME), the total time that the mice were in the dark box for activity or retention. We define the black box as a protected area (protected: p) and a non-protected area or an exposed area (unprotected: u). In this experiment, the retention time of the bright box was selected as an index to evaluate whether the mice had anxiety behavior.

### Western blotting

Twenty-four hours after last behavior test, the animals were sacrificed by decapitation and brain tissue was dissected. About 50 mg of brain tissue was homogenized in a homogenizer containing 400 ul of lysate and 10 ul of PMSF. After being lysed for 30 min on ice, the samples were centrifuged at 12,000 r/min at 4 °C for 15 min, the supernatant was separated. Protein concentration was determined using a BCA protein assay kit (Pierce Biotechnology, Inc, Rockford, USA). The same amount of protein was separated by electrophoresis and then transferred onto the PVDF membranes (Millipore). After sealing with 5% nonfat milk in Tris-buffered saline containing 0.1% Tween-20 (TBST) for 1 h at room temperature and rinsing, different primary antibodies (anti-NLRP1, anti-caspase-1, 1:800 dilution; anti-ASC, 1:200 dilution) were used to incubate the PVDF membranes overnight at 4 °C in a refrigerator. After rinsing, the membranes were added with horseradish peroxidase-conjugated secondary antibodies (1:10,000 dilution) in TBST with 1% nonfat milk for 1 h at room temperature. The membranes were reacted with enhanced chemiluminescence reagents (Amersham Pharmacia Biotech, Inc., Piscataway, NJ, USA) for 5 min and were visualized using chemiluminescence detection system (Bioshine, Shanghai, China).

### Quantitative real-time PCR analysis

Twenty-four hours after last behavior tests, the mice were sacrificed by decapitation and total RNA was extracted from brain tissue using TRIzol reagent (Invitrogen, USA) according to the manufacturer’s instructions. Using PrimeScript First Strand cDNA Synthesis Kit (Takara Biotechnology), we performed cDNA synthesis. The cDNA was amplified by PCR using standard methods. The following specific primers were used: NLRP1 (forward: 5-GCCCTGGAGACAAAGAATCC-3, reverse: 5-AGTGGGCATCGTCAT GTGT-3); ASC (forward: 5-ACCCCATAGACCTCACTGAT-3, reverse: 5-ACAGCT CCAGACTCTTCCAT-3); Caspase-1 (forward: 5-ATGCCGTGGAGAGAAACAAG-3, reverse: 5-CCAGGACACATTATCTGGTG-3); CXCR2 (forward:5-GTGATGCT GGTCATCTTGTAC-3 ,reverse:5-GCAGAACAGCATGATGAGCAG-3).CXCL1(forward:5-CGAGCCACACTCCAACACAGC-3, reverse:5-AGGGAGCTTCAGGGTCAAGGC-3). β-actin (forward: 5-TTCCTTCCTGGGTATGGAAT-3, reverse: 5-GAGG AGCAATGATCTTGATC-3). Each group was performed in triplicate, and the β-actin was used as internal reference. The fluorescent signals were collected during extension stage, Ct values of the sample were calculated and data were analyzed using the 2^−ΔΔCT^ method.

### Enzyme-linked immunosorbent assay (ELISA)

Twenty-four hours after last behavior test, the mice were sacrificed by decapitation and brain tissue was dissected. Protein samples were extracted and protein concentration determined as described above. The mean absorbance was determined for each group of duplicate standards and samples. The levels of inflammatory cytokines IL-1β, IL-18, IL-6, and TNF-α in brain tissue were measured by commercial ELISA kits (R&D Systems, Minneapolis, MN) according to the manufacturer’s protocols. The levels of cytokines IL-1β, IL-6, and TNF-α were expressed as pg/ml of the total protein.

### Injection of adenovirus-associated vector (AAV)

To study the role of NLRP1 inflammasome in stress-induced depressive-like behavior, adeno-associated virus (AAV) vectors containing Nlrp1a-shRNA or control-shRNA (Hanbio, Shanghai, China) was employed. In brief, Nlrp1a-shRNA or control-shRNA was cloned into pHBAAV-U6-MCS-CMV-eGFP (AAV2/9, 1.0 × 10^12^ TU/ml) and confirmed by sequencing. The recombinant plasmids were treated using a triple-transfection, helper-free method, and purified. The sequences for scrambled control-shRNA and Nlrp1a-shRNA were 5′-TTCTCCGAACGTGTCACGTAA-3′ and 5′-CAGCTAGA GAGGAACTTGAAGCTAA-3′ respectively. For the virus infusion, animals were anesthetized with isoflurane and 2 μl control-shRNA or Nlrp1a-shRNA were injected into the hippocampus (− 1.6 mm anteroposterior (AP), ±1.8 mm mediolateral (ML), − 1.6 mm dorsoventral (DV)) of mice (6 weeks old) at a rate of 0.2 μl/min using a 5-μl Hamilton syringe connected to a 30-gauge needle. Then, the animals were returned to their home cages while being kept warm. After 4 weeks period of recovery, fluorescence microscopy, western blotting, and qPCR were used to observe the effect of transfection in vivo and the stress procedures were performed.

### Statistical analysis

All data were analyzed with the statistical program SPSS 17.0 (Chicago, IL, USA). Data are expressed as means ± SEM. Statistical comparisons between experimental groups and control group were performed by using one-way analysis of variance (ANOVA) with an additional Bonferroni post hoc test. *P* < 0.05 was considered statistically significant.

## Results

### Chronic stress activates hippocampal NLRP1 inflammasome in mice

**Fig. 1:**
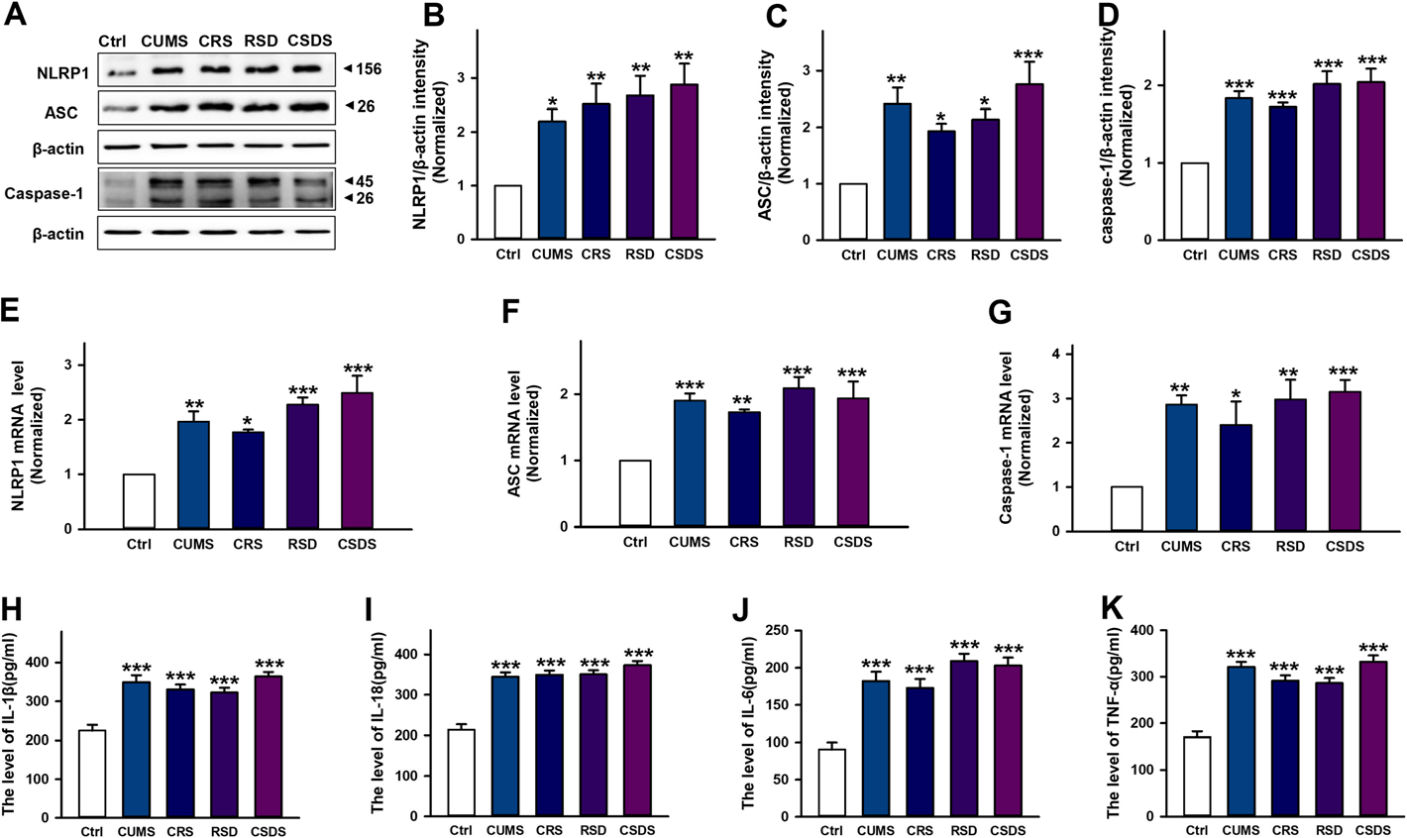
Chronic stress increases the expression of NLRP1 inflammasome complexes and pro-inflammatory cytokines levels in mice. **a** Representative immunoreactive bands showing the protein levels of hippocampal NLRP1, ASC and caspase-1 in the control, CUMS, CRS, RSDS, and CSDS mice. **b**–**d** statistical results show that CUMS, CRS, RSDS, and CSDS increased the protein expression of **b** NLRP1 (*n* = 6, *F*_4,25_ = 5.70, ^*^P < 0.05, ^**^P < 0.01 *vs* control), **c** ASC (*n* = 6, *F*_4,25_ = 7.39, ^*^P < 0.05, ^**^P < 0.01, ^***^P < 0.001 *vs* control) and **d** caspase-1 (*n* = 6, *F*_4,25_ = 13.0, ^***^P <0.001 *vs* control) in the hippocampus. **e**–**g** Statistical results show that CUMS, CRS, RSDS, and CSDS increased the mRNA expression of **e** NLRP1 (*n* = 6, *F*_4,25_ = 10.9, ^*^P < 0.05, ^**^P < 0.01, ^***^P < 0.001 *vs* control), **f** ASC (*n* = 6, *F*_4,25_ = 8.65, ^**^P < 0.01, ^***^P < 0.001 *vs* control) and **g** caspase-1 (*n* = 6, *F*_4,25_ = 6.33, ^*^P < 0.05, ^**^P < 0.01, ^***^P < 0.001 *vs* control) in the hippocampus. **h**–**k** Statistical results show that CUMS, CRS, RSDS, and CSDS increased the levels of **h** IL-1β (*n* = 6, *F*_4,25_ = 15.4, ^***^P < 0.001 *vs* control), **i** IL-18 (*n* = 6, *F*_4,25_ = 32.3, ^***^P < 0.001 *vs* control), **j** IL-6 (*n* = 6, *F*_4,25_ = 18.5, ^***^P < 0.001 *vs* control), and **k** TNF-α (*n* = 6, *F*_4,25_ = 29.1, ^***^P < 0.001 *vs* control) in the hippocampus. Data are expressed as means ± SEM. One-way ANOVA, Bonferroni test

To investigate the role of NLRP1 inflammasome in depression, we first established animal models by four chronic stimuli including CUMS, CRS, RSDS, and CSDS. Then, we tested the expression of hippocampal NLRP1 inflammasome complexes by western blot and RT-PCR. Our data showed that stress stimuli significantly increased the protein expression of NLRP1, ASC, and caspase-1 (Fig. 1a–d), and also markedly increased the mRNA levels of NLRP1, ASC, and caspase-1 (Fig. 1e–g), indicating NLRP1 inflammasome was activated in stress-induced depression models. Additionally, our data also showed that stress stimuli dramatically increased the level of pro-inflammatory cytokines such as IL-1β, IL-18, IL-6, and TNF-α (Fig. 1h–k) in the hippocampus. These results indicate that chronic stress activates NLRP1 inflammasome-inflammatory signaling in depressive-like mice.

### Hippocampal Nlrp1a knockdown ameliorates chronic stress induced depressive-like behaviors in mice

**Fig. 2:**
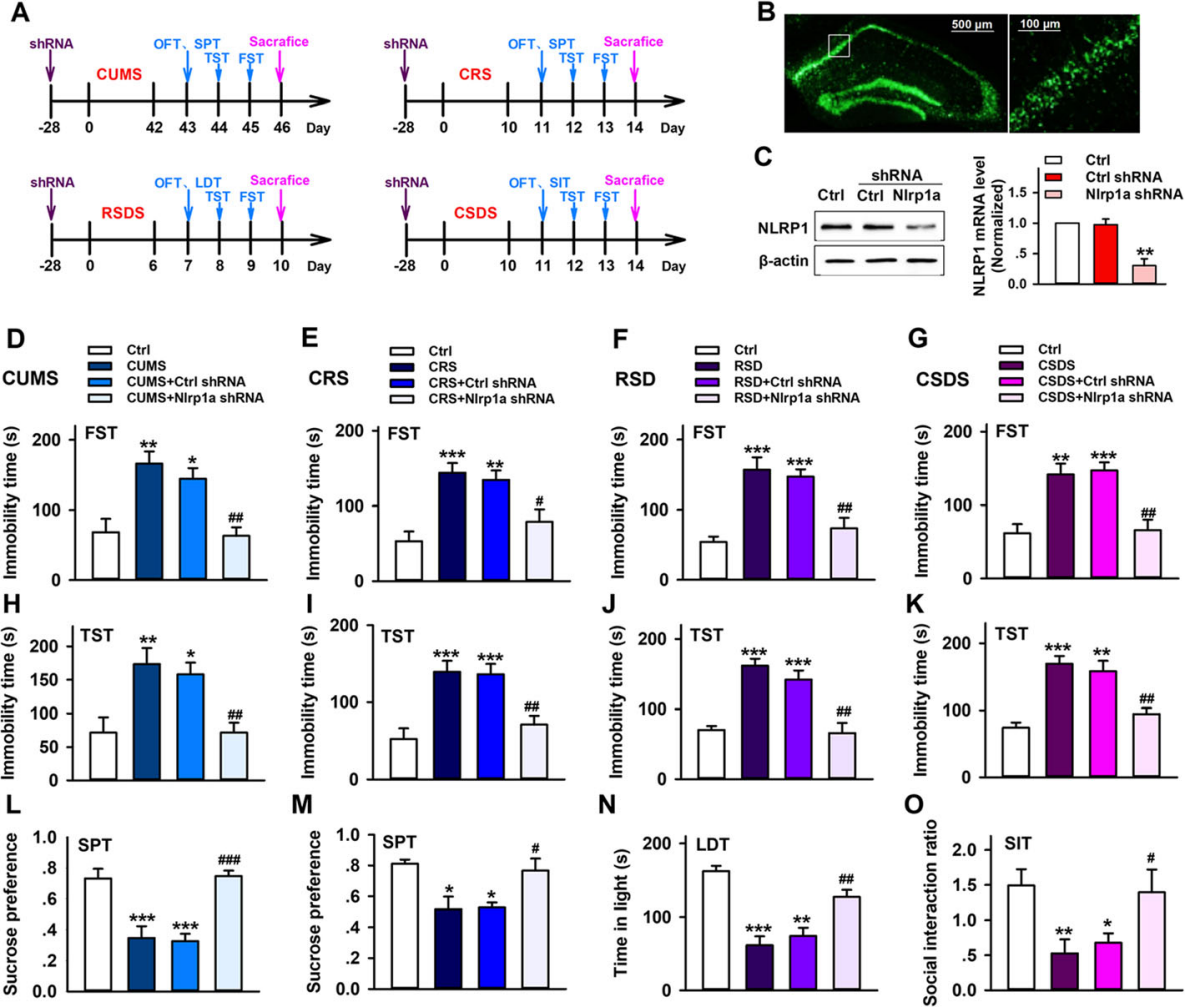
Hippocampal Nlrp1a knockdown ameliorates chronic stress-induced depressive-like behaviors in mice. **a** The scheme of AAV-shRNA infusion, stress stimuli and behavior test. Two micro liters control-shRNA or Nlrp1a-shRNA was injected into the hippocampus. Twenty-eight days after injection, the mice were exposed to CUMS (6 weeks), CRS (10 days), RSDS (6 days), and CSDS (10 days), respectively. Then FST, TST, and SPT were performed in CUMS and CRS mice. FST, TST, and LDT were performed in RSDS mice. FST, TST, and SIT were performed in CSDS mice. **b** Fluorescence images that expressed AAV-Nlrp1a-shRNA-EGFP in the hippocampus 28 days after infusion. Scale bar = 500 μm (left) and 100 μm (right). **c** Representative immunoreactive bands and the quantification of NLRP1 mRNA showing the knockdown efficacy of AAV-Nlrp1a-shRNA (*n* = 3, *F*_2,6_ = 22.0, ^**^P < 0.01 *vs* control and control shRNA). **d**–**g** Statistical results show that chronic stress increased immobility time in mice during FST, which has positive correction with depressive-like behavior, while Nlrp1a knockdown reserved the effect in **d** CUMS (*n* = 8, *F*_3,28_ = 10.4, ^*^P < 0.05, ^**^P < 0.01 *vs* control, ^##^*P* < 0.01 *vs* CUMS and control shRNA), **e** CRS (*n* = 8, *F*_3,28_ = 10.0, ^**^P < 0.01, ^***^P < 0.001 *vs* control, ^#^*P* < 0.05 *vs* CRS and control shRNA), **f** RSDS (*n* = 8, *F*_3,28_ = 15.1 ^***^P < 0.001 *vs* control, ^##^*P* < 0.01 *vs* RSDS and control shRNA), and **g** CSDS (*n* = 8, *F*_3,28_ = 12.2, ^**^P < 0.01, ^***^P < 0.001 *vs* control, ^##^*P* < 0.01 *vs* CSDS and control shRNA) mice. **h**–**k** Statistical results show that chronic stress increased immobility time in mice during TST, which has positive correction with depressive-like behavior, while Nlrp1a knockdown reserved the effect in **h** CUMS (*n* = 8, *F*_3,28_ = 7.31, ^*^P < 0.05, ^**^P < 0.01 *vs* control, ^##^*P* < 0.01 *vs* CUMS and control shRNA), **i** CRS (*n* = 8, *F*_3,28_ = 11.3 ^***^P<0.001 *vs* control, ^##^*P*<0.01 *vs* CRS and control shRNA), **j** RSDS (*n* = 8, *F*_3,28_ = 18.9, ^***^P < 0.001 *vs* control, ^##^*P* < 0.01 *vs* RSDS and control shRNA) and **k** CSDS (*n* = 8, *F*_3,28_ = 16.8, ^***^P < 0.001 *vs* control, ^##^*P* < 0.01 *vs* CSDS and control shRNA) mice. **l**, **m** Statistical results show that chronic stress decreased sucrose performance in mice during SPT, which has negative correction with depressive-like behavior, while Nlrp1a knockdown reserved the effect in **l** CUMS (*n* = 8, *F*_3,28_ = 16.1, ^***^P < 0.001 *vs* control, ^##^*P* < 0.01 *vs* CUMS and control shRNA) and **m** CRS (*n* = 8, *F*_3,28_ = 6.59, ^*^P < 0.05 *vs* control, ^#^*P* < 0.05 *vs* CRS and control shRNA) mice. **n** Statistical results show that chronic stress decreased the time in light during LDT, which has negative correction with depressive-like behavior, while Nlrp1a knockdown reserved the effect in RSDS mice (*n* = 8, *F*_3,28_ = 20.8, ^**^P < 0.01, ^***^P < 0.001 *vs* control, ^##^*P* < 0.01 *vs* RSDS and control shRNA). **o** Statistical results show that chronic stress decreased the social interaction during SIT, which has negative correction with depressive-like behavior, while Nlrp1a knockdown reserved the effect in CSDS mice (*n* = 8, *F*_3,28_ = 6.54, ^*^P < 0.05, ^**^P < 0.01 *vs* control, ^#^*P* < 0.05 *vs* CSDS and control shRNA). Data are expressed as means ± SEM. One-way ANOVA, Bonferroni test

To further study the potential role of NLRP1 inflammasome in depression, an adeno-associated virus (AAV) vector that selectively expresses Nlrp1a–shRNA with enhanced green fluorescent protein (AAV-Nlrp1a-shRNA-eGFP) was injected into the hippocampus of mice. As shown in Fig. 2b, c, Nlrp1a-shRNA showed clear silencing efficacy 4 weeks after AAV-shRNA infusion. Then CUMS, CRS, RSDS and CSDS were performed in these mice. After stress stimuli, depressive-like behavior was tested by FST, TST, SPT, LDT, and SIT (Fig. 2a). As shown in Fig. 2d–g, compared with control groups, all of four different chronic stresses induced significant increase in immobility time during FST. Nlrp1a knockdown reserved the effects, while control shRNA infusion has no influence on it. Similarly, Nlrp1a knockdown inhibited stress-induced increase in immobility time during TST (Fig. 2h–k). Also, Nlrp1a knockdown improved low ratio of sucrose preference induced by CUMS and CRS (Fig. 2l, m). Moreover, compared with control animals, RSDS led to significant decrease in the time spent in light (Fig. 2n), and CSDS resulted in markedly low social interaction ratio in susceptible mice (Fig. 2o). The effects can be reserved after Nlrp1a knockdown, while control shRNA infusion has no influence on them. These findings suggested that NLRP1inflammasome is involved in chronic stress-induced depressive-like behavior in mice.

### Hippocampal Nlrp1a knockdown prevents chronic stress induced decrease in mice locomotor activity

**Fig. 3:**
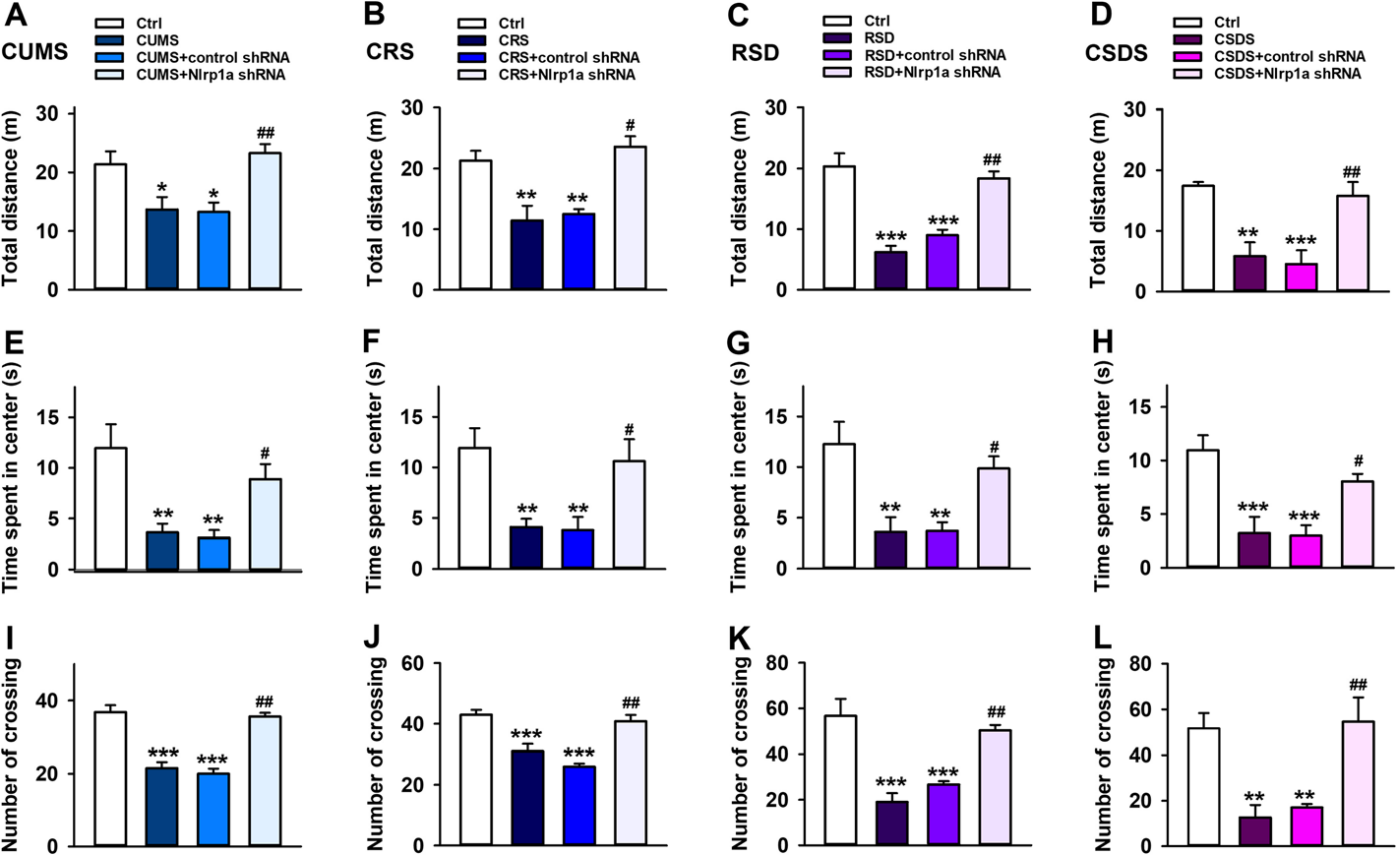
Effects of hippocampal Nlrp1a knockdown on the locomotor activity in chronic stress-induced depressive-like mice. **a**–**d** Statistical results show that chronic stress decreased the total moving distance in mice during OFT, which has negative correction with locomotor activity, while Nlrp1a knockdown reserved the effect in **a** CUMS (*n* = 8, *F*_3,28_ = 7.39, ^*^P < 0.05 *vs* control, ^##^*P* < 0.01 *vs* CUMS and control shRNA ), **b** CRS (*n* = 8, *F*_3,28_ = 12.5, ^**^P < 0.01 *vs* control, ^#^*P* < 0.05 *vs* CRS and control shRNA), **c** RSDS (*n* = 8, *F*_3,28_ = 23.9, ^***^P < 0.001 *vs* control, ^##^*P* < 0.01 *vs* RSDS and control shRNA), and **d** CSDS (*n* = 8, *F*_3,28_ = 11.1, ^**^P < 0.01, ^***^P < 0.001 *vs* control, ^##^*P* < 0.01 *vs* CSDS and control shRNA) mice. **e**–**h** Statistical results show that chronic stress decreased the time spent in center during OPT, which has negative correction with locomotor activity, while Nlrp1a knockdown reserved the effect in **e** CUMS (*n* = 8, *F*_3,28_ = 8.90, ^**^P < 0.01 *vs* control, ^#^*P* < 0.05 *vs* CUMS and control shRNA), **f** CRS (*n* = 8, *F*_3,28_ = 6.63, ^**^P < 0.01 *vs* control, ^#^*P* < 0.05 *vs* CRS and control shRNA), **g** RSDS (*n* = 8, *F*_3,28_ = 8.37, ^**^P < 0.01 *vs* control, ^#^*P* < 0.05 *vs* RSDS and control shRNA), and **h** CSDS (*n* = 8, *F*_3,28_ = 10.4, ^***^P < 0.001 *vs* control, ^#^*P* < 0.05 *vs* CSDS and control shRNA) mice. **i**–**l** Statistical results show that chronic stress decreased the numbers of crossing in mice during OPT, which has negative correction with locomotor activity, while Nlrp1a knockdown reserved the effect in **i** CUMS (*n* = 8, *F*_3,28_ = 32.7, ^***^P < 0.001 *vs* control, ^##^*P* < 0.01 *vs* CUMS and control shRNA), **j** CRS (*n* = 8, *F*_3,28_ = 18.5, ^***^P < 0.001 *vs* control, ^##^*P* < 0.01 *vs* CRS and control shRNA), **k** RSDS (*n* = 8, *F*_3,28_ = 16.6, ^***^P < 0.001 *vs* control, ^##^*P* < 0.01 *vs* RSDS and control shRNA), and **l** CSDS (*n* = 8, *F*_3,28_ = 10.2, ^**^P < 0.01 *vs* control, ^##^*P*<0.01 *vs* CSDS and control shRNA) mice. Data are expressed as means ± SEM. One-way ANOVA, Bonferroni test

Then, to study the effect of NLRP1 inflammasome on locomotor activity in mice with stress stimuli, CUMS, CRS, RSDS, and CSDS were performed 4 weeks after control shRNA or Nlrp1a shRNA infusion. OFT was used to evaluate the locomotor activity in mice with stress stimuli. As shown in Fig. 3a, e, and i, compared with control groups, CUMS caused significant decrease in total moving distance, time spent in center and the numbers of crossing. The effects were reversed by Nlrp1a shRNA infusion, while control shRNA infusion has no influence on locomotor activity in mice. Similar phenomenon was also observed in CRS (Fig.3b, f, j), RSDS (Fig. 3c, g, k), and CSDS (Fig. 3d, h, and l) models. These results suggested that NLRP1 inflammasome is implicated in chronic stress induced decrease in locomotor activity of mice.

### Hippocampal Nlrp1a shRNA infusion blocks NLRP1 inflammasome activation and inflammatory response in mice with stress stimuli

**Fig. 4:**
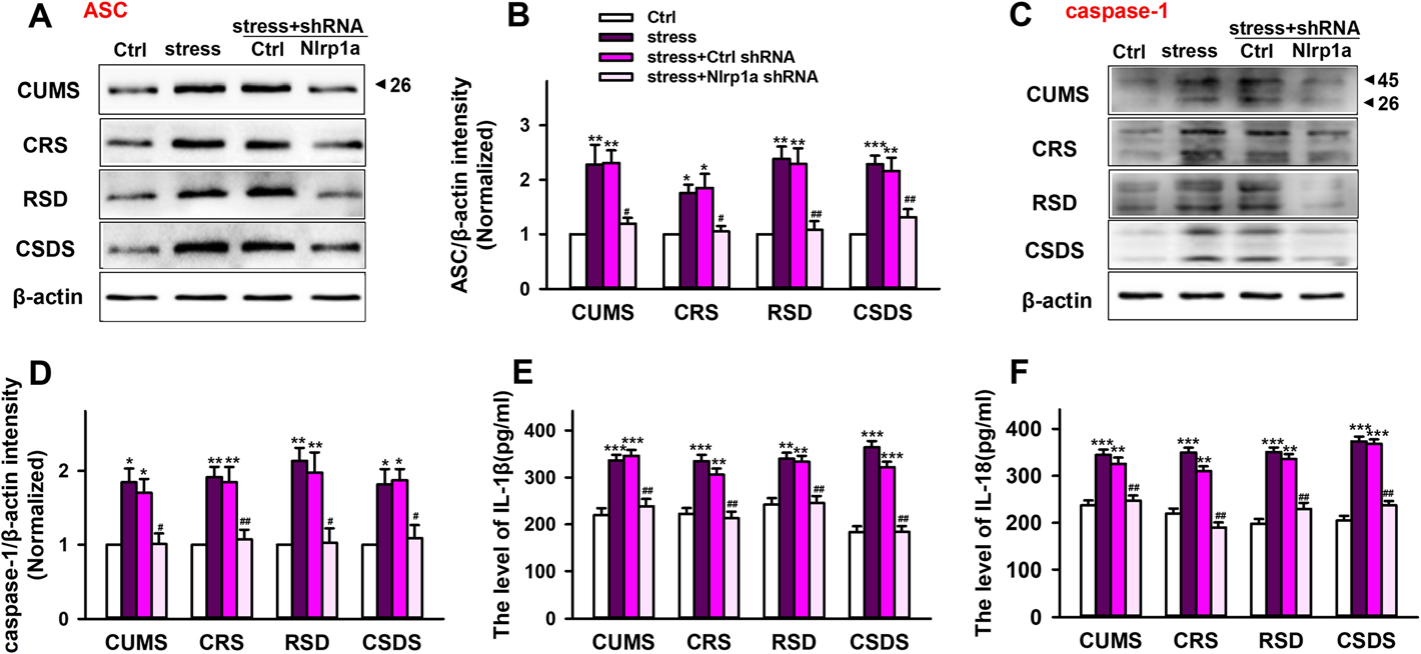
Nlrp1a knockdown blocks NLRP1 inflammasome activation in mice with stress stimuli. **a** Representative immunoreactive bands showing the protein levels of hippocampal ASC in the control, stress, control shRNA + stress, and Nlrp1a shRNA + stress mice. **b** Statistical results show that chronic stress increased the protein expression of ASC in hippocampus, while Nlrp1a knockdown inhibited the effects in CUMS (*n* = 4, *F*_3,12_ = 9.88, ^**^P < 0.01 *vs* control, ^#^*P* < 0.05 *vs* CUMS and control shRNA), CRS (*n* = 4, *F*_3,12_ = 8.10, ^*^P < 0.05 *vs* control, ^#^*P* < 0.05 *vs* CRS and control shRNA), RSDS (*n* = 4, *F*_3,12_ = 14.4, ^**^P<0.01 *vs* control, ^##^*P* < 0.01 *vs* RSDS and control shRNA), and CSDS (*n* = 4, *F*_3,12_ = 15.0, ^**^P < 0.01, ^***^P < 0.001 *vs* control, ^##^*P* < 0.01 *vs* CSDS and control shRNA) mice. **c** Representative immunoreactive bands showing the protein levels of hippocampal caspase-1 in the control, stress, control shRNA + stress, and Nlrp1a shRNA + stress mice. **d** Statistical results show that chronic stress increased the protein expression of caspase-1 in hippocampus, while Nlrp1a knockdown inhibited the effects in CUMS (*n* = 4, *F*_3,12_ = 8.84, ^*^P < 0.05 *vs* control, ^#^*P* < 0.05 *vs* CUMS and control shRNA), CRS (*n* = 4, *F*_3,12_ = 12.3, ^**^P < 0.01 *vs* control, ^##^*P* < 0.01 *vs* CRS and control shRNA), RSDS (*n* = 4, *F*_3,12_ = 10.2, ^**^P < 0.01 *vs* control, ^#^*P* < 0.05 *vs* RSDS and control shRNA), and CSDS (*n* = 4, *F*_3,12_ = 8.71, ^*^P < 0.05 *vs* control, ^#^*P* < 0.05 *vs* CSDS and control shRNA) mice. **e** Statistical results show that chronic stress increased the level of IL-1β in hippocampus, while Nlrp1a knockdown inhibited the effects in CUMS (*n* = 4, *F*_3,12_ = 22.5, ^***^P < 0.001 *vs* control, ^##^*P*<0.01 *vs* CUMS and control shRNA), CRS (*n* = 4, *F*_3,12_ = 20.2, ^**^P < 0.01, ^***^P < 0.001 *vs* control, ^##^*P* < 0.01 *vs* CRS and control shRNA), RSDS (*n* = 4, *F*_3,12_ = 15.3, ^**^P < 0.01 *vs* control, ^##^*P* < 0.01 *vs* RSDS and control shRNA) and CSDS (*n* = 4, *F*_3,12_ = 57.5, ^***^P < 0.001 *vs* control, ^##^*P* < 0.01 *vs* CSDS and control shRNA) mice. **f** Statistical results show that chronic stress increased the level of IL-18 in hippocampus, while Nlrp1a knockdown inhibited the effects in CUMS (*n* = 4, *F*_3,12_ = 21.5, ^**^P < 0.01, ^***^P < 0.001 *vs* control, ^##^*P* < 0.01 *vs* CUMS and control shRNA), CRS (*n* = 4, *F*_3,12_ = 51.5, ^**^P < 0.01, ^***^P < 0.001 *vs* control, ^##^*P* < 0.01 *vs* CRS and control shRNA), RSDS (*n* = 4, *F*_3,12_ = 50.4, ^**^P < 0.01, ^***^P < 0.001 *vs* control, ^##^*P* < 0.01 *vs* RSDS and control shRNA), and CSDS (*n* = 4, *F*_3,12_ = 79.7, ^***^P < 0.001 *vs* control, ^##^*P* < 0.01 *vs* CSDS and control shRNA) mice. Stress: CUMS, CRS, RSDS, and CSDS. Data are expressed as means ± SEM. One-way ANOVA, Bonferroni test.

The role of inflammation in depression has been addressed well. As a critical regulation platform of inflammatory response, the activation of inflammasome can promote the release of pro-inflammatory cytokines. To investigate the mechanism of NLRP1 inflammasome implicated in chronic stress induced depressive-like behavior, we test the hippocampal pro-inflammatory cytokines levels in mice. As shown in Fig. 4a–d, compared with control groups, all of four different chronic stresses induced significant increase in the expression of hippocampal ASC and caspase-1. While hippocampal Nlrp1a shRNA infusion inhibited the effects and successfully prevented NLRP1 inflammasome assembly. Moreover, hippocampal Nlrp1a shRNA infusion also decreased chronic stress induced increase in the level of pro-inflammatory cytokines IL-1β and IL-18 (Fig. 4e, f). These results suggested that NLRP1 inflammasome-mediated inflammatory response is responsible for chronic stress-induced depressive-like behavior in mice.

### Hippocampal Nlrp1a knockdown prevents the downregulation of BDNF levels induced by chronic stress in mice

**Fig. 5:**
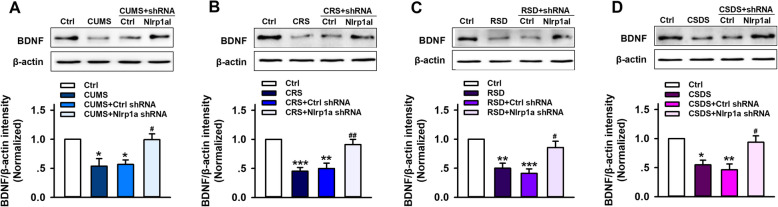
Effects of hippocampal Nlrp1a knockdown on BDNF levels in chronic stress-induced depressive-like mice. Representative immunoreactive bands and statistical results show that chronic stress decreased the protein expression of BDNF in hippocampus, while Nlrp1a knockdown restored BDNF expression in **a** CUMS mice (*n* = 4, *F*_3,12_ = 8.03, ^*^P < 0.05 *vs* control, ^#^*P* < 0.05 *vs* CUMS and control shRNA), **b** CRS mice (*n* = 4, *F*_3,12_ = 15.4, ^**^P < 0.01, ^***^P < 0.001 *vs* control, ^##^*P* < 0.01 *vs* CRS and control shRNA), **c** RSDS mice (*n* = 4, *F*_3,12_ = 12.7, ^**^P < 0.01, ^***^P < 0.001 *vs* control, ^#^*P* < 0.05 *vs* RSDS and control shRNA) and **d** CSDS mice (*n* = 4, *F*_3,12_ = 10.2, ^*^P < 0.05, ^**^P < 0.01 *vs* control, ^#^*P* < 0.05 *vs* CSDS and control shRNA). Data are expressed as means ± SEM. One-way ANOVA, Bonferroni test

BDNF, one of the major neurotrophic factors, plays a role in the pathophysiology of depression [39, 40]. However, little is known about the correlation between NLRP1 inflammasome and BDNF signal in stress-induced depressive-like behavior. To determine the issue, the mice received control shRNA or Nlrp1a shRNA infusion at 28 days before stress stimuli (CUMS, CRS, RSDS, and CSDS) as previous description. After stress process, the protein expression of BDNF was tested. As shown in Fig. 5, compared with control groups, all four stress stimuli-induced significant decrease in the protein expression of hippocampal BDNF. Nlrp1a shRNA infusion can inhibit the effects and restore BDNF levels, while control shRNA infusion has no influence on them. This result indicates that NLRP1-BDNF signaling mediates stress-induced depressive-like behavior in mice.

### Hippocampal Nlrp1a knockdown prevents the upregulation of CXCL1/CXCR2 signal induced by chronic stress in mice

**Fig. 6:**
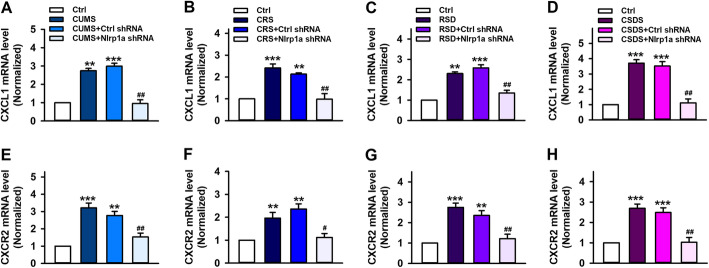
Effects of hippocampal Nlrp1a knockdown on the expression of CXCL1/CXCR2 in chronic stress-induced depressive-like mice. **a**–**d** Statistical results show that chronic stress increased the mRNA expression of CXCL1 in hippocampus, while Nlrp1a knockdown inhibited the effects in **a** CUMS mice (*n* = 4, *F*_3,12_ = 59.0, ^**^P < 0.01, ^***^P < 0.001 *vs* control, ^##^*P* < 0.01 *vs* CUMS and control shRNA), **b** CRS mice (*n* = 4, *F*_3,12_ = 21.9, ^**^P < 0.01, ^***^P < 0.001 *vs* control, ^##^*P* < 0.01 *vs* CRS and control shRNA), **c** RSDS mice (*n* = 4, *F*_3,12_ = 47.1, ^**^P < 0.01, ^***^P < 0.001 *vs* control, ^##^*P* < 0.01 *vs* RSDS and control shRNA) and **d** CSDS mice (*n* = 4, *F*_3,12_ = 44.3, ^***^P < 0.001 *vs* control, ^##^*P* < 0.01 *vs* CSDS and control shRNA). **e**–**h** Statistical results show that chronic stress increased the mRNA expression of CXCR2 in hippocampus, while Nlrp1a knockdown inhibited the effects in **e** CUMS mice (*n* = 4, *F*_3,12_ = 23.2, ^**^P < 0.01, ^***^P < 0.001 *vs* control, ^##^*P* < 0.01 *vs* CUMS and control shRNA), **f** CRS mice (*n* = 4, *F*_3,12_ = 12.4, ^**^P < 0.01 *vs* control, ^#^*P* < 0.05 *vs* CRS and control shRNA), **g** RSDS mice (*n* = 4, *F*_3,12_ = 20.1, ^**^P < 0.01, ^***^P < 0.001 *vs* control, ^##^*P* < 0.01 *vs* RSDS and control shRNA), and **h** CSDS mice (*n* = 4, *F*_3,12_ = 22.1, ^***^P < 0.001 *vs* control, ^##^*P*<0.01 *vs* CSDS and control shRNA). Data are expressed as means ± SEM. One-way ANOVA, Bonferroni test

CXCL1, one of the major chemoattractants for neutrophils, and its receptor CXCR2 have been reported to involve in CUMS-induced depressive-like behavior in mice [41]. However, the correlation between NLRP1 inflammasome and CXCL1/CXCR2 signal in stress-induced depressive-like behavior remains unclear. To address this issue, control shRNA or Nlrp1a shRNA was infused to hippocampus of mice at 28 days before stress stimuli. After stress process, the mRNA expression of CXCL1 and CXCR2 were tested. As shown in Fig. 6a–e, compared with control groups, the expression of hippocampal CXCL1 and CXCR2 is significantly increased in CUMS-induced depressive-like mice. It is consistent with previous report [41]. Meanwhile, the expression of CXCL1 and CXCR2 is also increased in depressive-like mice induced by CRS, RSDS, and CSDS (Fig. 6b–d, f–h). Interestingly, Nlrp1a knockdown reserved the upregulation of CXCL1/CXCR2 in these mice treated with CUMS, CRS, RSDS, and CSDS, while control shRNA infusion has no influence on them. Taken together, these results indicate that NLRP1-CXCL1/CXCR2 signaling mediated chronic stress-induced depressive-like behavior in mice.

## Discussion

In the present study, we demonstrated that NLRP1 inflammasome and related inflammatory signaling are activated in chronic stress-induced animal model of depression. Blockage of NLRP1 inflammasome-driven inflammatory signaling ameliorates depressive-like behaviors of the animals and reserves the up-regulation of hippocampal CXCL1/CXCR2 signaling and the down-regulation of BDNF signaling induced by stress stimuli, suggesting NLRP1 inflammasome is involved in the processes of MDD through regulating CXCL1/CXCR2-BDNF signaling pathway.

Neuroinflammation, which is an innate immune response to tissue damage, plays an important role in many CNS disorders including psychiatric illnesses and is believed to contribute to the initiation, relapse, and progression of MDD [42–44]. Inflammasomes, a key component of the innate immune response, have been reported to implicate in the mechanism of inflammation related neurological disorders [27]. NLRP1 is the first to be characterized inflammasome and can be activated by several stimuli, including *Bacillus anthracis* lethal toxin, *Toxoplasma gondii*, muramyl dipeptide, host intracellular ATP depletion, low [K^+^]_i_ and extracellular acidosis [44, 45]. Activated NLRP1 inflammasome cleave pro-caspase-1 into active capsase-1, which subsequently results in the production of pro-inflammatory cytokines IL-1β and IL-18, thereby triggering the inflammatory response [46]. NLRP1 inflammasome-driven inflammatory pathway has been reported to implicate in many neurological diseases such as brain injury, neurodegenerative diseases, nociception, and epilepsy [46, 47]. However, little is known about the role of NLRP1 inflammasome in depression. Our previous study showed that chronic glucocorticoid exposure activated NLRP1 inflammasome in the brain, whereas chronic glucocorticoid treatment has been used to induce animal depressive-like state [48]. Therefore, we propose that NLRP1 inflammasome could play an important role in depression. To test this hypothesis, we first established depression models of mice via four different stress stimuli: CUMS, CRS, CSDS and RSDS. We found that the expression of hippocampal NLRP1, ASC, and caspase-1 increase in different animal models. Similarly, the levels of hippocampal pro-inflammatory cytokines (IL-1β, IL-18, IL-6, and TNF-α) also increase in these models. These indicate that stress stimuli lead to the assembly of NLRP1 inflammasome and consequently triggers inflammatory response, which probably contributes to the development of depression. To further determine the speculation, AAV-Nlrp1a-shRNA was infused to hippocampus of mice, which results in Nlrp1a silencing efficacy at 4 weeks after infusion. Interestingly, hippocampal Nlrp1a knockdown almost reserved chronic stress-induced depressive-like behaviors and improved the spontaneous motor activity in mice. Also, Nlrp1a-shRNA infusion prevented the assembly of NLRP1 inflammasome and consequently inflammatory responses, while control shRNA itself has no effects on inflammasome complexes and animal’s behavior (Fig. 2c and Additional file 1). These indicate that inflammatory pathology in hippocampus is critical for depression. Moreover, attenuation of hippocampal neurogenesis has been suggested as the potential etiology for depression [49]. It also is an important target of most antidepressant treatments [50]. In addition, hippocampus contains high levels of glucocorticoid receptors and glutamate and regulates the hypothalamus-pituitary-adrenal (HPA) axis, which makes it is particularly sensitive to stress [51, 52]. Thus, stress-induced inflammatory process may occur in hippocampus prior to other brain regions. Inhibition of inflammatory pathology in hippocampus could be critical for preventing inflammatory process in the brain. However, we do not exclude the role of inflammatory pathology in other brain region in depression. Maybe hippocampal inflammatory process is more important in the development of depression. Taken together, NLRP1 inflammasome-driven inflammatory response in hippocampus is crucial for stress-induced depressive-like behaviors.

BDNF belongs to the family of neurotrophins and plays critical role in survival, development, and maintenance of nervous [53, 54]. Many studies have shown that BDNF is implicated in the pathophysiology of depression and antidepressant efficacy [39, 40]. Brain BDNF levels have been found to be reduced in animals and patients with depression, as well as postmortem samples of subjects with depression [54–57]. Chronic antidepressant treatment can increase BDNF expression in hippocampus and prefrontal cortex. Moreover, central administration BDNF may reduce depressive symptoms, while conditional BDNF knockout in animals produces depression-like effects [58–60]. Additionally, several studies have demonstrated that administration of LPS or pro-inflammatory cytokine significantly reduced BDNF levels in hippocampus and cerebral cortex [61]. That indicates that inflammation affects the expression of BDNF, which contributes to the effect of inflammation on the development of depression. Our results have displayed that NLRP1 inflammasome-driven inflammatory response is implicated in depression. Thus, NLRP1 inflammasome could affect the levels of BDNF in stress-induced depression model. To demonstrate this hypothesis, we test the expression of hippocampal BDNF in depressive-like mice. We found that chronic stress stimuli caused low BDNF levels in four different animal models, which is consistent with previous reports [55, 57]. Interestingly, hippocampal NLRP1a knockdown reversed chronic stress-induced decrease in the expression of BDNF. This result suggests that activated NLRP1 inflammasome down-regulated BDNF signal, which could be responsible for its role in the development of depression.

Chemokines are small chemotactic cytokines which can induce chemotaxis and migration of immune cells and play an important role in neurogenesis, neuron-glia communication, synaptic transmission and plasticity [62, 63]. CXCL1, a C-X-C chemokine family member, is a chemotactic cytokine produced during inflammation and is responsible for attracting polymorphonuclear cells towards the inflammatory site [62]. Previous studies indicated that CXCL1 might participate in the pathogenesis of depression [63]. The expression of CXCL1 is increased in interferon α induced depressive-like mice [64]. Also, RSDS led to an increase in the levels of CXCL1 in the brain of mice, and CXCL1 chemokines treatment caused rat depressive-like behaviors [65, 66]. Recent study showed that CXCL1 and its receptor CXCR2 are upregulated in CUMS induced depressive-like mice. CXCL1 overexpression in hippocampus induced depressive-like behavior and decreased BDNF levels, whereas CXCR2 inhibition blocked depressive-like behavior and restored BDNF levels [41]. Combined with our previous results, we think that chronic stress activates NLRP1 inflammasome and consequently triggers inflammatory response resulting in production of CXCL1 and causes the down-regulation of BDNF, which could contribute to depressive-like behaviors in mice. To test this hypothesis, we knocked down hippocampal Nlrp1a and test the changes of CXCL1/CXCR2 expression in the hippocampus. We found that CXCL1 and CXCR2 mRNA levels are upregulated in four different stress models, and Nlrp1a knockdown inhibited the upregulation of CXCL1/CXCR2 expression, indicating that CXCL1/CXCR2 signaling mediated BDNF downregulation could contribute to the effect of NLRP1 inflammasome on depressive-like behaviors. It should be noted that the mice were not perfused before collecting samples in the present study. To test whether perfusion or no perfusion affects the experimental results, we compared the data from perfused samples and no perfused samples under normal condition, stress stimuli and Nlrp1a shRNA treatment in western bot, qPCR, and ELISA test. Our results showed that the mean value of the data in no perfusion groups seem to be higher than that in perfusion groups, but the results of statistical analyze showed that there is no significant difference between them (Additional file 2), indicating that no perfusion could has a little effect on the data, but it did not affect the final results. Also, the precise mechanism of NLRP1 inflammasome implicating in stress-induced depressive-like behaviors was not be specifically demonstrated. Therefore, more effort should be done in the future.

## Conclusions

The present study demonstrated that NLRP1 inflammasome-driven inflammatory process is a critical player in chronic stress-induced depression-like behaviors. These effects could be mediated by CXCL1/CXCR2/BDNF signaling pathway. Therefore, NLRP1 inflammasome is a potential antidepressant target and inhibition of NLRP1 inflammasome can protect against chronic stress-induced depression-like behaviors.

## Supplementary information

**Additional file 1: Figure S1.** The effect of Ctrl shRNA on inflammasome complexes and behavior in mice. (A) and (B) Statistical results show that Ctrl shRNA treatment has no influence on the expression of hippocampal ASC (A) and caspase-1(B) in mRNA levels. n=4. (C) and (D) Statistical results show that Ctrl shRNA treatment did not affect the immobility time of mice in FST (C) and TST(D). n=6. (E) and (H) Statistical results show that Ctrl shRNA treatment has no effect on the sucrose preference, total distance, time in light and social interaction ratio in SPT, OFT, LDT and SIT respectively. n=6. Data are expressed as means ± SEM, statistical analyze was performed by using two-sided and unpaired Student’s t-test.

**Additional file 2: Figure S2.** The effect of perfusion or no perfusion on the expression of inflammasome complexes and the levels of IL-1β and CXCL1. (A) Representative immunoreactive bands and statistical results show that Nlrp1a shRNA treatment significantly inhibited CUMS-induced increase in the protein expression of hippocampal ASC in perfusion (Per) brain and no perfusion (NP) brain. (B) Statistical results show that Nlrp1a shRNA treatment significantly inhibited CRS-induced increase in the mRNA levels of hippocampal ASC in perfusion (Per) brain and no perfusion (NP) brain. (C) Statistical results show that Nlrp1a shRNA treatment significantly inhibited RSD-induced increase in the levels of hippocampal IL-1β in perfusion (Per) brain and no perfusion (NP) brain. (D) Statistical results show that Nlrp1a shRNA treatment significantly inhibited CSDS-induced increase in the mRNA levels of hippocampal CXCL1 in perfusion (Per) brain and no perfusion (NP) brain. Although the mean value of the data in no perfusion groups seem to be higher than that in perfusion groups, the results of statistical analyze showed that there is no significant difference between perfusion brain and no perfusion brain. Data are expressed as means ± SEM, n=6, statistical analyze was performed by using two-away ANOVA with Bonferroni post hoc test. **P<0.01 *vs* control, ## *P*<0.01 vs CUMS, CRS, RSD or CSDS.

## Data Availability

The datasets used and/or analyzed during the current study are available from the corresponding author on reasonable request.

## Abbreviations

AAV: Adeno-associated virus;;
ASC: Apoptosis-associated speck-like protein containing a caspase-activating recruitment domain
CRS: Chronic restrain stress
CSDS: Chronic social defeat stress
CUMS: Chronic unpredictable mild stress
ELISA: Enzyme-linked immunosorbent assay
FST: Forced swim test
LDT: Light-dark test
MDD: Major depressive disorder
NLRP1: Nucleotide oligomerization domain (NOD)-like receptor protein 1
OFT: Open-field test
RSD: Repeat social defeat
SIT: Social interaction test
SPT: Sucrose preference test
TST: Tail-suspension test
