# Supplementary material for: NLRP1 inflammasome contributes to chronic stress-induced depressive-like behaviors in mice
Source: J Neuroinflammation. 2020 Jun 8;17:178. doi: 10.1186/s12974-020-01848-8 (PMC7281929; doi:10.1186/s12974-020-01848-8)
Supplement: Supplementary file 1 — Additional file 1: Figure S1. The effect of Ctrl shRNA on inflammasome complexes and behavior in mice. (A) and (B) Statistical results show that Ctrl shRNA treatment has no influence on the expression of hippocampal ASC (A) and caspase-1(B) in mRNA levels. n=4. (C) and (D) Statistical results show that Ctrl shRNA treatment did not affect the immobility time of mice in FST (C) and TST(D). n=6. (E) and (H) Statistical results show that Ctrl shRNA treatment has no effect on the sucrose preference, total distance, time in light and social interaction ratio in SPT, OFT, LDT and SIT respectively. n=6. Data are expressed as means ± SEM, statistical analyze was performed by using two-sided and unpaired Student’s t-test. [file 12974_2020_1848_MOESM1_ESM.pdf]

Figure S1

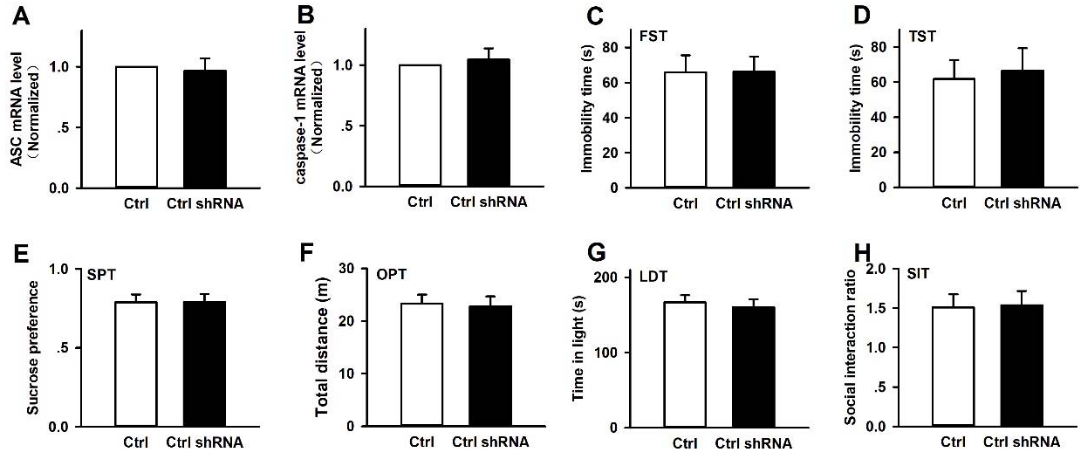

Fig. S1 The effect of Ctrl shRNA on inflammasome complexes and behavior in mice. (A) and (B) Statistical results show that Ctrl shRNA treatment has no influence on the expression of hippocampal ASC (A) and caspase-1(B) in mRNA levels. n=4. (C) and (D) Statistical results show that Ctrl shRNA treatment did not affect the immobility time of mice in FST (C) and TST(D). n=6. (E) and (H) Statistical results show that Ctrl shRNA treatment has no effect on the sucrose preference, total distance, time in light and social interaction ratio in SPT, OPT, LDT and SIT respectively. n=6. Data are expressed as means ± SEM, statistical analyze was performed by using two-sided and unpaired Student's t-test.
