# Supplementary material for: NLRP1 inflammasome contributes to chronic stress-induced depressive-like behaviors in mice
Source: J Neuroinflammation. 2020 Jun 8;17:178. doi: 10.1186/s12974-020-01848-8 (PMC7281929; doi:10.1186/s12974-020-01848-8)
Supplement: Supplementary file 2 — Additional file 2: Figure S2. The effect of perfusion or no perfusion on the expression of inflammasome complexes and the levels of IL-1β and CXCL1. (A) Representative immunoreactive bands and statistical results show that Nlrp1a shRNA treatment significantly inhibited CUMS-induced increase in the protein expression of hippocampal ASC in perfusion (Per) brain and no perfusion (NP) brain. (B) Statistical results show that Nlrp1a shRNA treatment significantly inhibited CRS-induced increase in the mRNA levels of hippocampal ASC in perfusion (Per) brain and no perfusion (NP) brain. (C) Statistical results show that Nlrp1a shRNA treatment significantly inhibited RSD-induced increase in the levels of hippocampal IL-1β in perfusion (Per) brain and no perfusion (NP) brain. (D) Statistical results show that Nlrp1a shRNA treatment significantly inhibited CSDS-induced increase in the mRNA levels of hippocampal CXCL1 in perfusion (Per) brain and no perfusion (NP) brain. Although the mean value of the data in no perfusion groups seem to be higher than that in perfusion groups, the results of statistical analyze showed that there is no significant difference between perfusion brain and no perfusion brain. Data are expressed as means ± SEM, n=6, statistical analyze was performed by using two-away ANOVA with Bonferroni post hoc test. **P<0.01 vs control, ## P<0.01 vs CUMS, CRS, RSD or CSDS. [file 12974_2020_1848_MOESM2_ESM.pdf]

Figure S2

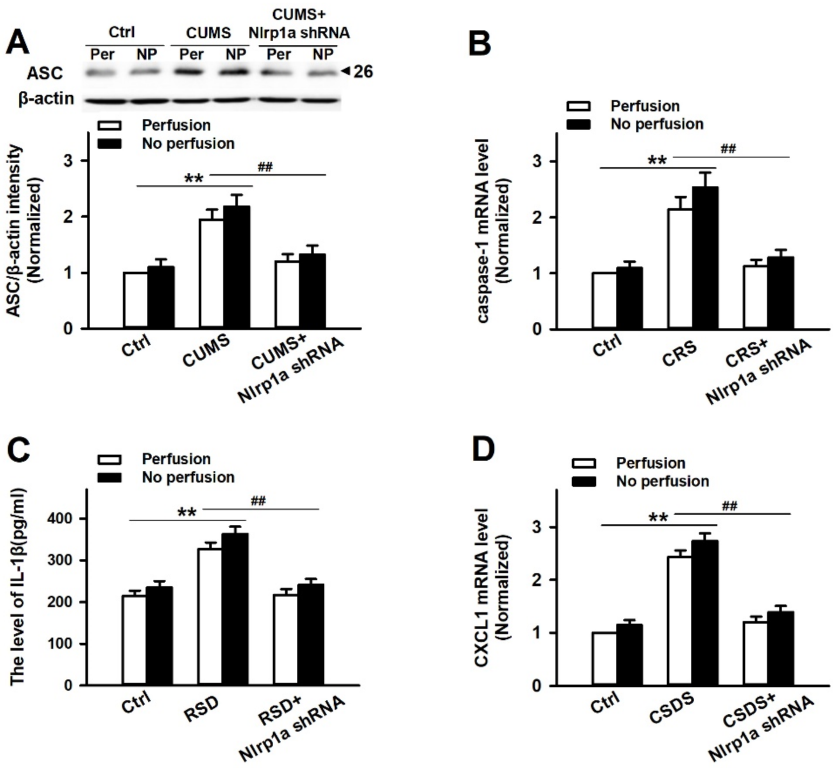

Fig. S2 The effect of perfusion or no perfusion on the expression of inflammasome complexes and the levels of IL-1β and CXCL1. (A) Representative immunoreactive bands and statistical results show that Nlrp1a shRNA treatment significantly inhibited CUMS-induced increase in the protein expression of hippocampal ASC in perfusion (Per) brain and no perfusion (NP) brain. (B) Statistical results show that Nlrp1a shRNA treatment significantly inhibited CRS-induced increase in the mRNA levels of hippocampal ASC in perfusion (Per) brain and no perfusion (NP) brain. (C) Statistical results show that Nlrp1a shRNA treatment significantly inhibited RSD-induced increase in the levels of hippocampal IL-1β in perfusion (Per) brain and no perfusion (NP) brain. (D) Statistical results show that Nlrp1a shRNA treatment significantly inhibited CSDS-induced increase in the mRNA levels of hippocampal CXCL1 in perfusion (Per) brain and no perfusion (NP) brain. Although the mean value of the data in no perfusion groups seem to be higher than that in perfusion groups, the results of statistical analyze showed that there is no significant difference between perfusion brain and no perfusion brain. Data are expressed as means ± SEM, n=6, statistical analyze

was performed by using two-away ANOVA with Bonferroni post hoc test. $^{**}P<0.01$ *vs* control, $^{\#\#}P<0.01$ vs CUMS, CRS, RSD or CSDS.
